# Combination of Arsenic and Interferon-α Inhibits Expression of KSHV Latent Transcripts and Synergistically Improves Survival of Mice with Primary Effusion Lymphomas

**DOI:** 10.1371/journal.pone.0079474

**Published:** 2013-11-08

**Authors:** Hiba El Hajj, Jihane Ali, Akram Ghantous, Dana Hodroj, Ahmad Daher, Kazem Zibara, Chloé Journo, Zaher Otrock, Ghazi Zaatari, Renaud Mahieux, Marwan El Sabban, Ali Bazarbachi, Raghida Abou Merhi

**Affiliations:** 1 Department of Internal Medicine, Faculty of Medicine, American University of Beirut, Beirut, Lebanon; 2 Lebanese University, Rafik Hariri Campus, Faculty of Sciences, Biology Department, Hadath, Lebanon; 3 International Agency for Research on Cancer, Lyon, France; 4 Lebanese University, Faculty of Sciences, Biology Department, fifth section, Nabatieh, Lebanon; 5 Equipe Oncogenèse Rétrovirale, Equipe labelisée “Ligue Nationale Contre le Cancer” INSERM U1111 - CNRS UMR5308, CIRI - International Center for Infectiology Research, Biology Department, Ecole Normale Supérieure de Lyon, Lyon, France; 6 Leukemia Program, Cleveland Clinic Taussig Cancer Institute, Cleveland, Ohio, United States of America; 7 Department of Pathology and Laboratory Medicine, Faculty of Medicine, American University of Beirut, Beirut, Lebanon; 8 Department of Anatomy, Cell Biology and Physiology, Faculty of Medicine, American University of Beirut, Beirut, Lebanon; University of Southern California Keck School of Medicine, United States of America

## Abstract

**Background:**

Kaposi sarcoma-associated herpesvirus (KSHV) is the etiologic agent of primary effusion lymphomas (PEL). PEL cell lines infected with KSHV, but negative for Epstein-Barr virus have a tumorigenic potential in non-obese diabetic/severe combined immunodeficient mice and result in efficient engraftment and formation of malignant ascites with notable abdominal distension, consistent with the clinical manifestations of PEL in humans.

**Methodology/Principal Findings:**

Using this preclinical mouse model, we demonstrate that the combination of arsenic trioxide and interferon-alpha (IFN) inhibits proliferation, induces apoptosis and downregulates the latent viral transcripts LANA-1, v-FLIP and v-Cyc in PEL cells derived from malignant ascites. Furthermore, this combination decreases the peritoneal volume and synergistically increases survival of PEL mice.

**Conclusion/Significance:**

These results provide a promising rationale for the therapeutic use of arsenic/IFN in PEL patients.

## Introduction

Infection with Kaposi sarcoma associated herpesvirus (KSHV) (also known as Human Herpesvirus Type 8 (HHV-8)) [1], is linked to all forms of Kaposi sarcoma, primary effusion lymphoma (PEL) [2–4], and some forms of multicentric Castelman’s disease (MCD) [5,6]. PEL is a monoclonal/oligoclonal, rare, aggressive and body cavity-based B-cell lymphoma, accounting for approximately 3% of AIDS-related lymphomas [7,8]. This unusual lymphoproliferative disorder is divided into classical and solid variants. The classical PEL is characterized by malignant effusions in the serosal surfaces, mostly pleural, pericardial and peritoneal cavities and by the absence of an obvious tumor mass, lymphadenopathy or hepatosplenomegaly [9]. The solid PEL manifests with extracavitary tissue-based tumors that may precede PEL development [10], may follow malignant effusions [11], or may not at all be associated with PEL serous effusions [3,6,10,12–14]. The presence of KSHV genome in PEL cells, in addition to the fact that a number of KSHV encoded viral proteins possesses transforming ability [15], suggests that KSHV contributes to B-cell transformation [16,17]. KSHV genome encodes 80 open reading frames (ORFs) [18–20]. KSHV infection, similar to most herpesviruses, exhibits two different types of cycles: a latent and a lytic infection cycle. Generally, KSHV maintains a stringent latent infection, and it is thought that the oncopathology of KSHV is mainly due to the viral products produced during latency [7,21]. The main latent genes include the Latency Associated Nuclear Antigens LANA-1 and 2 [9,22], the viral cyclin (v-Cyc), and viral FLICE inhibitory protein (v-FLIP). LANA-1 [23] causes cell cycle progression, impairs apoptosis, and increases hypoxia inducible factor-1α (HIF-1α) levels, which leads to activation of genes involved in angiogenesis, cell proliferation, and survival [24]. LANA-2 antagonises p53-mediated apoptosis *in vitro* [25], and stimulates c-Myc [26]. V-Cyc, a viral homologue of cellular cyclin D, binds to human cyclin-dependent kinase 6 (CDK6) resulting in resistance to CDK inhibitors, progression through the cell cycle, and uncontrolled cell division [27]. V-Cyc may also lead to centrosomal abnormalities that contribute to malignant transformation through genomic instability [28]. Lastly, v-FLIP, a homologue of cellular FLIP, functions both as an inhibitor of death receptor mediated apoptosis and an activator of the transcription factor NF-κB [29]. Importantly, mice transgenic for LANA, v-FLIP, or v-Cyc develop lymphoid malignancies with low frequency and after a long latency [30–32].

PEL patients rarely respond to conventional systemic chemotherapy and their prognosis is poor, with a median survival of less than six months [17,22]. Several alternative treatments have now been tested in limited series of patients, including high-dose chemotherapy and autologous stem cell transplantation [22,33,34]. A chemotherapy regimen that includes high dose methotrexate was shown to induce complete remission in a number of AIDS-associated PEL patients [35]. Moreover, intra-pleural cidofovir showed some benefit in one patient [36]. In preclinical studies, a number of drugs were shown to induce apoptosis in KSHV-infected PEL cells [37–43]. Indeed, rapamycin (sirolimus) as well as the combination of interferon-α (IFN) and zidovudine (AZT) induce apoptosis in PEL cell lines and in NOD/SCID mice xenografts [44–47]. Finally, the current and most promising treatment strategies in PEL patients are directed towards combining the available anti-viral treatments with other agents including chemicals and cytokines.

Arsenic trioxide (arsenic) is a very effective treatment of acute promyelocytic leukemia (APL) [48–54]. Similarly, in human T cell leukemia virus type 1 (HTLV-1) associated adult T-cell leukemia (ATL) [55], we have shown that the combination of arsenic and IFN degrades the viral oncoprotein Tax, cures murine ATL and induces a high rate of response when combined with AZT in human chronic ATL [ 56–63]. Finally, in PEL cell lines, we have shown that the combination of arsenic and IFN inhibits growth and NF-κB activation and induces caspase-dependent apoptosis [64].

In this report, using the preclinical NOD/SCID mouse model, we demonstrate that the combination of arsenic and IFN inhibits proliferation, induces apoptosis and downregulates the latent viral transcripts LANA-1, v-FLIP and v-Cyc in PEL cells derived from malignant ascites. Furthermore, *in vivo* administration of this drug combination decreases the peritoneal volume and synergistically increases survival of PEL mice. Our results provide a promising rationale for the therapeutic use of arsenic/IFN in PEL patients. 

## Materials and Methods

### Cells, mice, and treatments

BC-1, BC-3 and BCBL-1 cell lines are KSHV^+^/EBV^-^ malignant B cells derived from PEL patients [19,65] and were obtained from American Type Culture Collection (Manassas, VA) or from Dr A. Gessain (Pasteur Institute, Paris, France). RAJI, BL-41 and Jurkat are KSHV^-^ malignant B (RAJI, BL41) and T (Jurkat) cells and were used as negative control. Cells were grown in RPMI-1640 medium containing 10% heat inactivated fetal calf serum and antibiotics. 

For *in vivo* experiments, we used the previously reported PEL-like mouse model [43]. Briefly, 2 million BC-3 or BCBL-1 cells were inoculated into the peritoneal cavities of 5 to 8 week old immuno-compromised NOD/SCID mice (Charles River, France). All murine protocols were approved by the Institutional Animal Care and Utilization Committee (IACUC) of the American University of Beirut (AUB). All animals were housed in specific pathogen-free facilities. Humane endpoints were used as requested by the AUB IACUC according to AAALAC (Association for Assessment and Accreditation of Laboratory Animal Care International) guidelines and guide of animal care use book (Guide, NRC 2011). Mice were sacrificed for any of the following reasons: 1) impaired mobility (the inability to reach food and water); 2) inability to remain upright; 3) clinical dehydration and/or prolonged decreased food intake; 4) weight loss of 15-20%; 5) self-mutilation; 6) lack of grooming behavior/rough/unkempt hair coat for more than 48 hours; 7) significant abdominal distension; 8) unconsciousness with no response to external stimuli. Animals were deeply anesthetized before cervical dislocation. 

Recombinant interferon-alpha (IFN) (Roferon®, Hoffman-La Roche) Azidothymidine (AZT) and arsenic trioxide (arsenic) were purchased from Sigma (Aldrich, France). For *in vivo* experiments, mice received arsenic (5 μg/g/day) intraperitoneally, IFN (10^5^ IU/day) subcutaneously, and/or AZT at 1.1 mg/day intraperitoneally. These doses are comparable with those used in other mouse models and predicted to yield plasma concentrations similar to those noted in patients [45,51,54,66]. Treatment was given for a total of 21 days as previously described for murine adult T cell leukemia model derived from Tax transgenic mice [63]. None of the individual or combination treatment regimen was toxic in normal NOD/SCID mice when given for 21 days (100% survival for >3 months; no observed acute or delayed toxicities; *n* = 3 for each condition). 

For *ex vivo* analysis, BC-3 and BCBL-1 malignant ascites were collected from PEL NOD/SCID mice after the development of lymphomatous effusions (4 weeks after injection). Cells were cultured at the density of 2 x 10^5^ cells per ml and were maintained in a humidified incubator at 37°C, 5% CO2. Drugs were added at the concentrations of 1 μM arsenic, 1000 IU/ml IFN, and 10 µg/ml AZT at the initiation of cultures for 24, 48, or 72 h. Cell growth was assessed by cell count using trypan blue dye exclusion protocols, and the CellTiter 96^®^ cell proliferation assay kit (Promega Corp., Madison, WI, USA). For dose-dependent experiments, cells were treated with 0.1, 0.5 or 1µM arsenic, alone or in combination with 1000 IU/ml IFN.

### Mice survival, phenotype and statistical analysis

Mice survival curves were calculated according to the method of Kaplan–Meier. Overall survival is defined as the time from injection of PEL cells to death from any cause. For phenotype analysis, PEL NOD/SCID mice were visually monitored and peritoneal diameter (d) was measured with a caliber to assess ascites development. Peritoneal volume was calculated according to the formula: v=4/3π(d/2)^3^ [67]. 

### Histopathology

Tissues from either treated or untreated mice were fixed in neutral buffer formalin (Sigma), embedded in paraffin, sectioned, stained with hematoxylin and eosin (H&E), and examined by light microscopy. 

### Annexin V staining

Phosphatidyl-serine (PS) exposure in *ex-vivo* treated PEL-derived cells was assessed using Annexin V-FITC kit (Immunotech/Beckman Coulter) according to manufacturer instructions. Approximately 10000 cells per sample were acquired and analyzed using the CellQuest software (Becton Dickinson). Similar experiments were performed on BL-41 and BC-1 cells, using Fixable Viability Stain 450 (BD Biosciences) and PE Annexin V (BD Biosciences) according to manufacturer instructions. Approximately 20000 cells per sample were acquired and analyzed using the FACSDiva software (BD Biosciences).

### TUNEL assay

*Ex-vivo* treated PEL-derived cells were rapidly collected and the assay was immediately performed according to the manufacturer's (Roche) recommendations. Fluorescein-conjugated dUTP incorporated in nucleotide polymers was detected and quantified using flow cytometry. Approximately 10000 cells per sample were acquired and analyzed using the CellQuest software (Becton Dickinson). 

### CD45 labeling

Cells were collected from untreated mice, washed twice with PBS, then incubated with human CD45 (Becton Dickinson) at the dilution 1 in 5. Approximately 10000 cells per sample were acquired using the FACScanto II machine and analyzed using the CellQuest software (Becton Dickinson).

### PCR and qRT- PCR

Tissues were harvested from PEL NOD/SCID mice having developed solid tumor mass. Regular PCR and real-time RT-PCR were used to quantitate the absolute LANA1, v-FLIP and v-Cyclin DNA content and mRNA expression, using a 2X PCR (Master Mix, Fermentas), and Light Cycler RNA Master kit (Roche Diagnostics), respectively. We also used the Lightcycler 4.05 software version. Primers sequences were designed by TIB-MOLBIOL (Germany): V-FLIP (Forward, 5’- gTgTTCATACCTCAACCCACAC; Reverse, 5’- CACACAgCTCCCCgTCTAC); V-cyclin (Forwad, 5’- TCAgTTTgCCAggAATACAACCTAg; Reverse, 5’- AAgAAggAAgTTACgTCCgTCg); LANA-1 (Forwad, 5’-CCgAggACgAAATggAAgTg; Reverse, 5’-ggTgATgTTCTgAgTACATAgCgg); ORF50/RTA (Forward: CgCAATgCgTTACgTTgTTg; Reverse: gCCCggACTgTTgAATCg). Normalization of the transcript levels was done with the G6PDH or beta actin housekeeping gene internal levels. 

### Immunocytochemistry and immunofluorescence assays

Cytospin preparations from untreated BC-3 and BCBL-1 ascites were performed on charged slides. Cells were fixed with ice-cold methanol for 5 minutes, and then washed with ice-cold PBS 1X. Permeabilization was achieved through incubation with PBS containing 0.25% triton 100X. For immunocytochemistry (ICC), the monoclonal rat anti LANA-1 (Abcam, UK, LN-35, 1:50) was added at dilution 1:25 in (PBS 1X, 1% BSA) for 90 mins at 37°C. After rinsing with PBS 1X, cells were incubated with peroxidase labeled anti-rat (Chemicon, AP136P, 1:200) for 90 minutes at 37°C. The same procedure was performed for immunofluorescence using the secondary anti-rat ﬂuorescein isothiocyanate (FITC) antibody (Abcam, ab 97056; 1:100). Then cells were washed with PBS and stained with 3,3’Diaminobenzidine (DAB) substrate chromogen system (Novocastra, Leica, DAB chromogen) (RE7105) and substrate buffer (RE7106). Images were taken using a Zeiss Axiocam Camera and software (Carl Zeiss, Thornwood, NY).

### Statistical Analysis

SPSS Version 16.0 and Microsoft Office Excel 2010 were used for statistical analyses. Except for survival analyses, mean values were reported and compared using ANOVA with associated *post-hoc* tests: Dunnett*t*, Tukey, and Student-Newman-Keuls (SNK). When applicable, SPSS syntaxtool was used to perform multiple comparisons within interaction groups in multi-Way ANOVA. In survival analyses, median values were reported and compared using Log Rank, Breslow, and Tarone-Ware tests, with similar results. Statistical significance was reported when the *p-value* was ≤ 0.05. The symbol *was used to compare treatment groups to control, while the symbol ‡was used to compare combination treatments to single treatments. (*,‡) indicates p< 0.05; (**,‡‡) indicates p< 0.01; and (***,‡‡‡) indicates p< 0.001.

## Results

### Arsenic and IFN delayed ascites formation and synergistically prolonged survival in PEL mice

**Figure 1:**
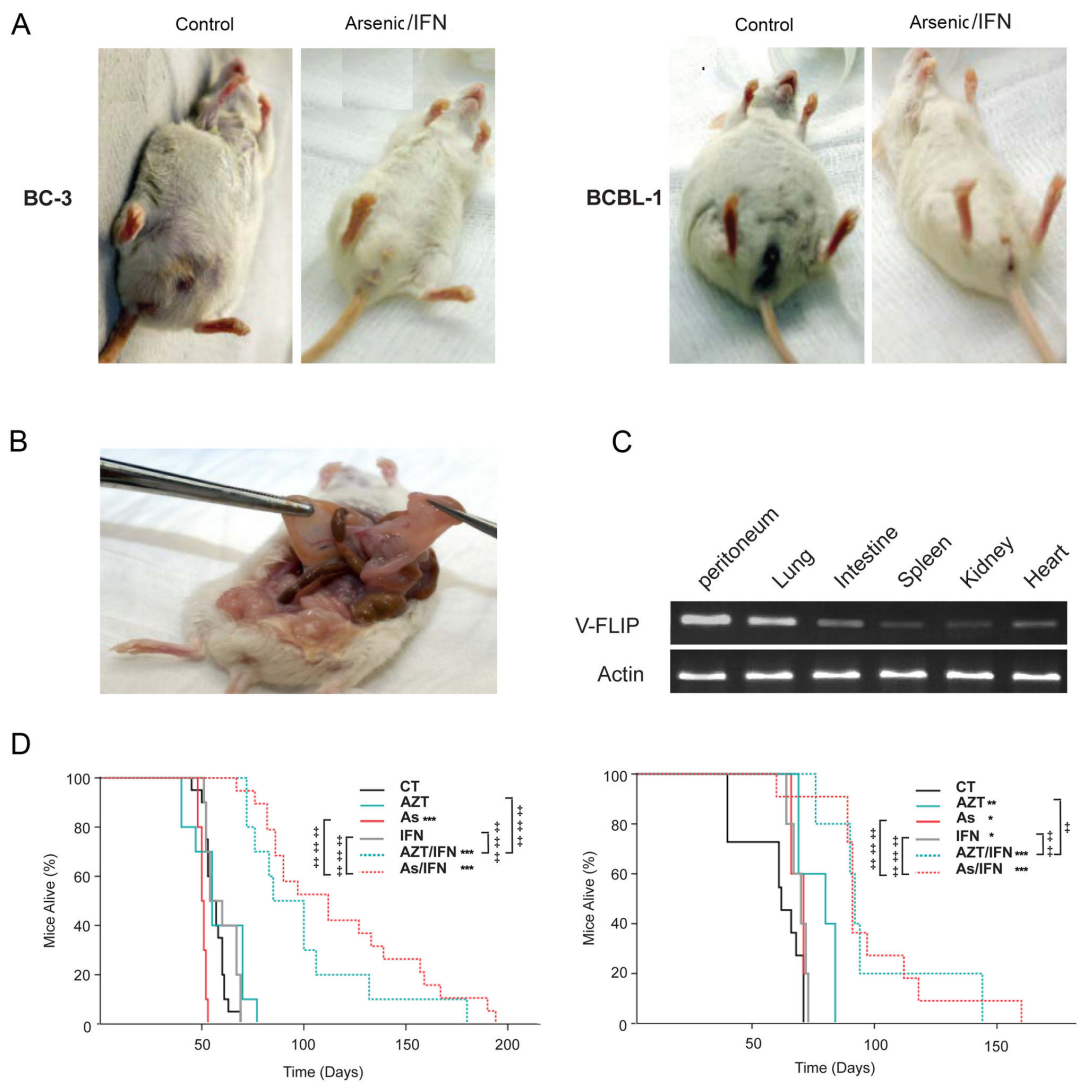
Arsenic and IFN synergistically prolonged survival in PEL mice. (**A**) Mice phenotype before and after treatment with arsenic/IFN. (**B**) Solid PEL tumors in untreated mice. (**C**) PCR for V-FLIP gene expression in different organs. (**D**) Kaplan–Meier analysis of overall survival curves of PEL NOD/SCID mice. Mice (*n* = 10 for each condition) were inoculated with 2x10^6^ of BC-3 (left) and BCBL-1 (right) cells, respectively. Treatment with the single agent drugs or their combinations was initiated at 2 days post-inoculation of PEL cells for a total of 21 days. The symbol * was used to compare treatment groups to control, while the symbol ‡ was used to compare combination treatments to single treatments. (*, ‡) indicates p< 0.05; (**, ‡‡) indicates p< 0.01; and (***, ‡‡‡) indicates p< 0.001.

BC-3 or BCBL-1 cells were inoculated intra-peritoneally into NOD/SCID mice. These cells showed efficient PEL engraftment reflected by the development of malignant ascites within 4-5 weeks post-inoculation. The gross anatomy of these PEL mice showed increase in the peritoneal volume, ascites formation or peritoneal solid tumor growth (Figure 1A, 1B). Histopathology examination and PCR analysis for v-FLIP revealed infiltration of the spleen, liver, lung and peritoneum by KSHV positive malignant cells (Figure 1C, Figure S1). These findings are consistent with the clinical manifestations of PEL in humans [65].

On day 2 post-inoculation of PEL cells, mice were treated with arsenic, IFN, AZT, or their combinations (arsenic/IFN or AZT/IFN) for a total of 21 days. When compared to untreated controls, a limited or no survival advantage was seen in mice receiving IFN, AZT or arsenic alone (Figure 1D), but a significant prolonged survival was observed in both BC3 and BCBL-1 mice when treated with the combination of arsenic/IFN or AZT/IFN (Figure 1D). Indeed, in the BC-3 model, median survival increased from 54 days in control to 85 and 112 days in mice treated with AZT/IFN or arsenic/IFN, respectively (p < 0.001 for both). Similarly, in the BCBL-1 model, median survival increased from 62 days in control to 92 and 91 days in AZT/IFN and arsenic/IFN treated mice, respectively (p < 0.001 for both). Statistical comparison between single agent therapy versus combination revealed a major synergy between arsenic and IFN in both BC-3 and BCBL-1 mice (p<0.001 for both) whereas AZT and IFN displayed a more synergistic effect in BC-3 mice (p<0.001) as compared to BCBL-1 mice (p<0.05) (Figure 1D). 

**Figure 2:**
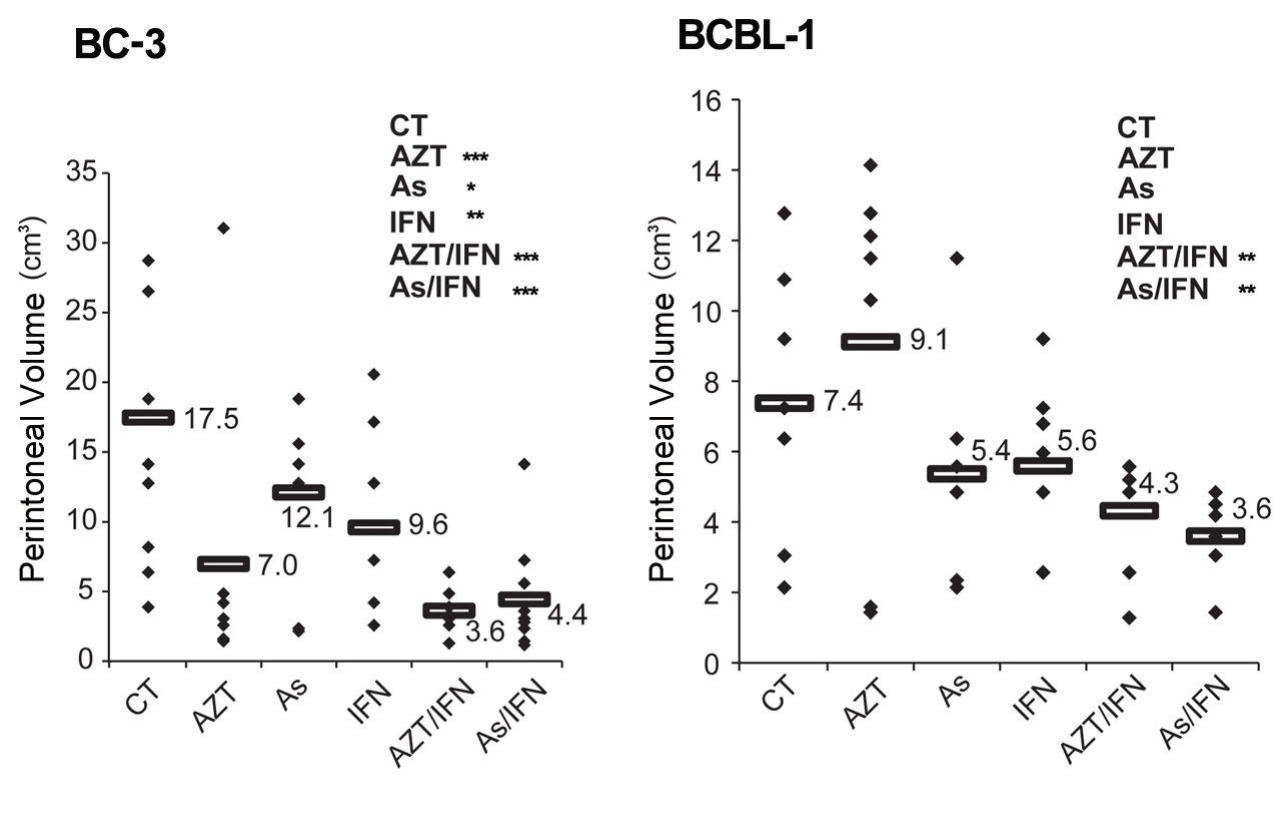
Arsenic and IFN delayed ascites formation in PEL mice. Peritoneal volume on day 45 post-treatment. PEL NOD/SCID mice (n=10 per condition) were visually monitored. Peritoneal diameter (d) was measured with a caliber to assess ascites development. Peritoneal volume was calculated according to the formula: v=4/3π(d/2)^3^. The symbol * was used to compare treatment groups to control (*, **, and *** indicates p< 0.05, p< 0.01, and p< 0.001, respectively).

Mice were also monitored for their peritoneal volume. Treatment with either arsenic/IFN or AZT/IFN delayed ascites formation and abdominal distention in PEL mice (Figure 1A). Indeed, in BC-3 mice, treatment with either arsenic/IFN or AZT/IFN significantly decreased the peritoneal volume 45 days post-inoculation of PEL cells, in comparison with untreated control: mean decreasing from 17.5 cm^3^ to 4.4 cm^3^ with arsenic/IFN (p<0.001) or to 3.3 cm^3^ with AZT/IFN (p<0.001) (Figure 2). Similarly, in BCBL-1 mice, mean peritoneal volume decreased from 7.4 cm^3^ to 3.6 cm^3^ with arsenic/IFN (p<0.01) and to 4.3 cm^3^ with AZT/IFN (p<0.01) (Figure 2). Single agent treatments resulted in significant decrease in peritoneal volume in BC3 but not BLBL-1 mice (Figure 2). 

### Arsenic/IFN synergistically inhibited proliferation and induced apoptosis of ascites-derived BC-3 and BCBL-1 cells

**Figure 3:**
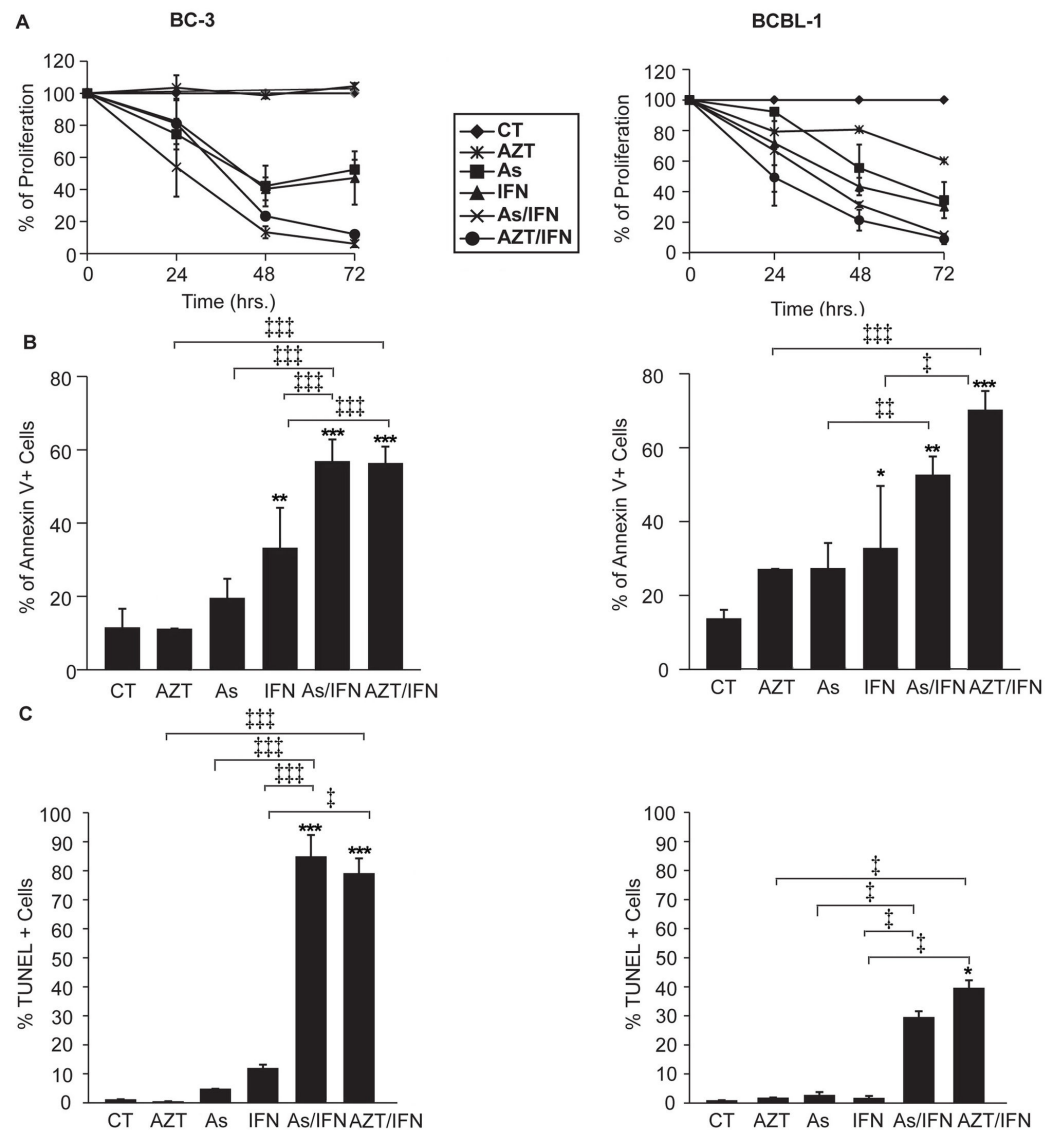
Arsenic/IFN synergistically inhibited proliferation and induced apoptosis of ascites-derived BC3 (left) and BCBL-1 cells (right). (**A**) Cell Proliferation: Cells were plated in a 96 well format and treated with the single agent drugs or their combinations for 24, 48, and 72h. Results are expressed as percent of control, plotted as mean ± SD, and are representative of two independent experiments. (**B**) Annexin V staining: BC-3 and BCBL-1 ascites were treated for 48h. Histograms represent the proportion of apoptotic cells. Results are plotted as mean ± SD and are representative of at least 2 independent experiments. (**C**) TUNEL assay: BC-3 and BCBL-1 cells derived from PEL ascites were treated for 72h. Histograms represent apoptotic cells as percentage of the untreated controls and are plotted as mean ± SD.

BC-3 and BCBL-1 cells derived from malignant ascites in PEL mice were *ex-vivo* treated with arsenic, IFN, AZT, or the combinations of arsenic/IFN or AZT/IFN up to 72h. A minimal to moderate inhibitory effect on cell proliferation was observed with single agent treatments, compared with AZT/IFN and arsenic/IFN, both of which showed synergistic effects (Figure 3A, p<0.05). 

To assess whether this synergy is arsenic dose-dependent and PEL-specific, different arsenic concentrations (0.1, 0.5 and 1 μM) were tested on both PEL-derived (BC-3, BCBL-1, BC-1) and KSHV negative cell lines (RAJI, BL-41 and Jurkat). Cell growth was assessed up to 72h post-treatment. Upon treatment with arsenic 0.5 or 1 μM, the three PEL-derived cell lines (BC-3, BCBL-1 and BC-1) underwent a significant drop in cell proliferation (Figure S2a). On the other hand, treatment with arsenic 0.1 μM or IFN alone had only a moderate inhibitory effect at all time points. Addition of IFN to arsenic (0.5 μM) and mostly arsenic (1 μM) resulted in additive to synergistic effect in PEL derived cell lines, especially BC-1 cells, whereas minimal effect was observed in the non-PEL cell lines (RAJI, BL-41 and Jurkat) (Figure S2a).

To assess whether the observed inhibition of ascites-derived BC-3 and BCBL-1 cell proliferation resulted from apoptosis, ex-vivo treated BC-3 and BCBL-1 cells were stained with Annexin V. A moderate increase in the apoptotic population was detected upon treatment with single agent arsenic or IFN but a major increase in Annexin V positive cells exceeding 50% at 48h was observed for both ascites-derived BC3 and BCBL-1 cells treated with either arsenic/IFN or AZT/IFN (p< 0.001 for both) (Figure 3B, Figure S3A). This result is consistent with our previous results on arsenic/IFN induced apoptosis on PEL cell lines [64]

To confirm that apoptosis is arsenic dose-dependent and PEL-specific, we compared Annexin V/Fixable Viability Stain positivity in PEL-derived (BC-1) and KSHV negative (BL-41) cell lines, upon treatment with IFN 1000 IU/ml and/or arsenic 0.1, 0.5 or 1 μM. Interestingly, arsenic and IFN synergized to induce apoptosis at 24h and more prominently at 48h in BC-1 cells whereas minimal effect was observed in BL-41 cells (Figure S2B).

**Figure 4:**
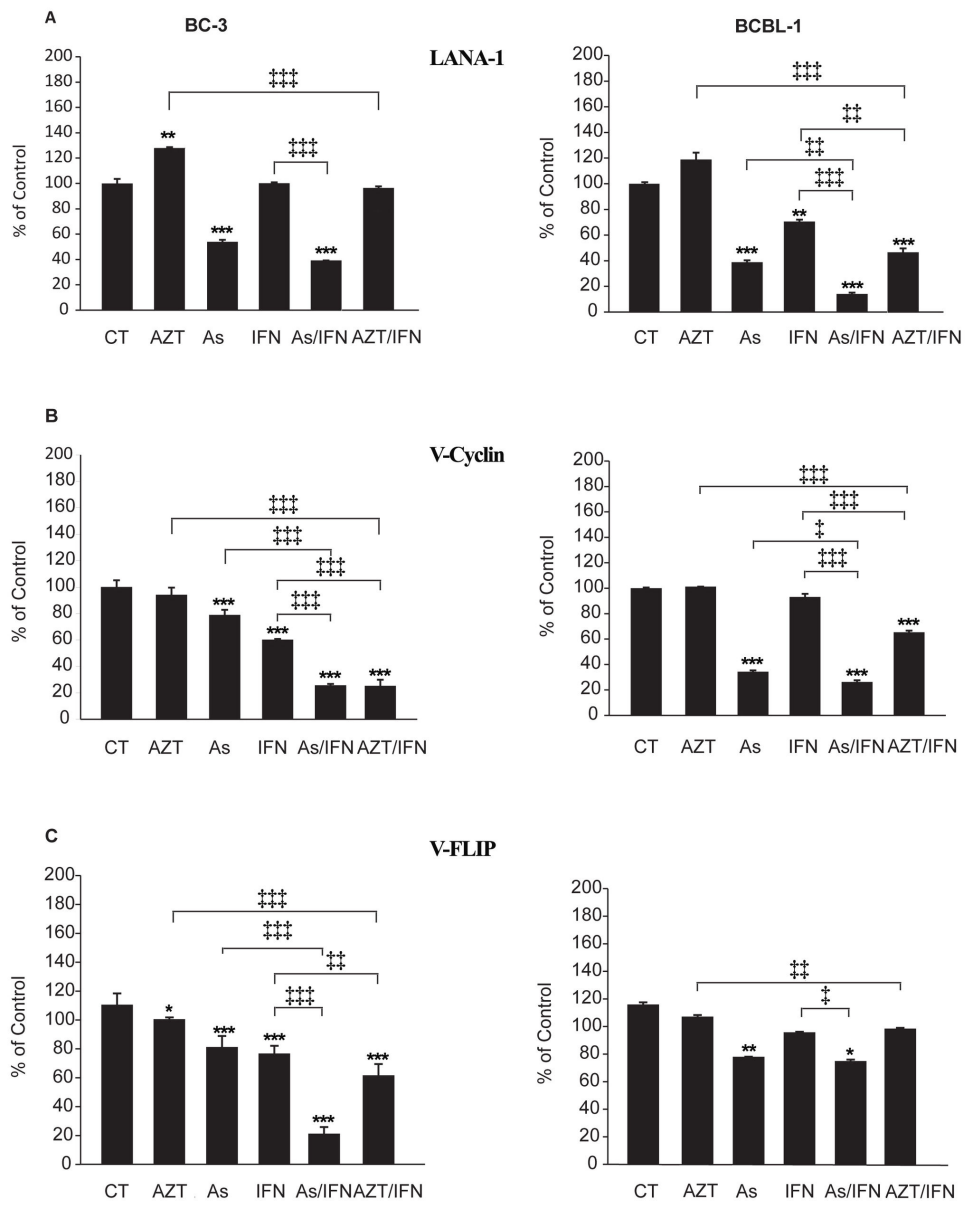
Arsenic combined with IFN induced capase-dependent apoptosis and latent viral proteins downregulation in BC-3 cells. (**A**) Western blot analysis of BC-3 cells treated for 48h using PARP-specific antibody. (**B**) Western blot analysis of *ex-vivo* treated (48h) ascites derived BC-3 cells using LANA-1 and LANA-2 specific antibodies.

Consistent with these results, a major increase in TUNEL positivity was observed for both ascites-derived BC-3 and BCBL-1 cells treated with arsenic/IFN or AZT/IFN combinations (p< 0.001), whereas minimal effects were seen with single agent treatments (Figure 3C, p< 0.05, Figure S3B). Finally, after 48 h, arsenic and IFN resulted in complete cleavage of PARP, indicative of caspase activation (Figure 4A). Collectively, these results indicate that arsenic or AZT synergize with IFN to induce apoptosis and inhibit proliferation of PEL cells derived from malignant ascites. 

### Arsenic/IFN inhibited expression of latent viral transcripts

To assess whether the therapeutic effect of arsenic/IFN correlates with modulation of expression of the KSHV latent transcripts, we tested the variation of LANA-1, v-FLIP, and v-Cyc mRNA in BC-3 and BCBL-1 cells derived from PEL ascites. Viral expression within ascitic cells was verified by western blot for LANA-1 and LANA-2 (Figure S4A), real-time RT-PCR for LANA-1 transcript (Figure S4B), ICC and IFA for LANA-1 (Figures S4C and S4D). Human CD45 staining (Figure S4E) clearly demonstrated that cells were of human and not murine origin. Finally, and in order to exclude a loss of episomes within some fraction of human cells over time in this model, we sequentially verified LANA-1 and LANA-2 protein levels (Figure S4A) and LANA-1 transcript levels (Figure S4B) at different time points in ex-vivo culture (day 0 till day 11). No significant and consistent variation was noted. 

**Figure 5:**
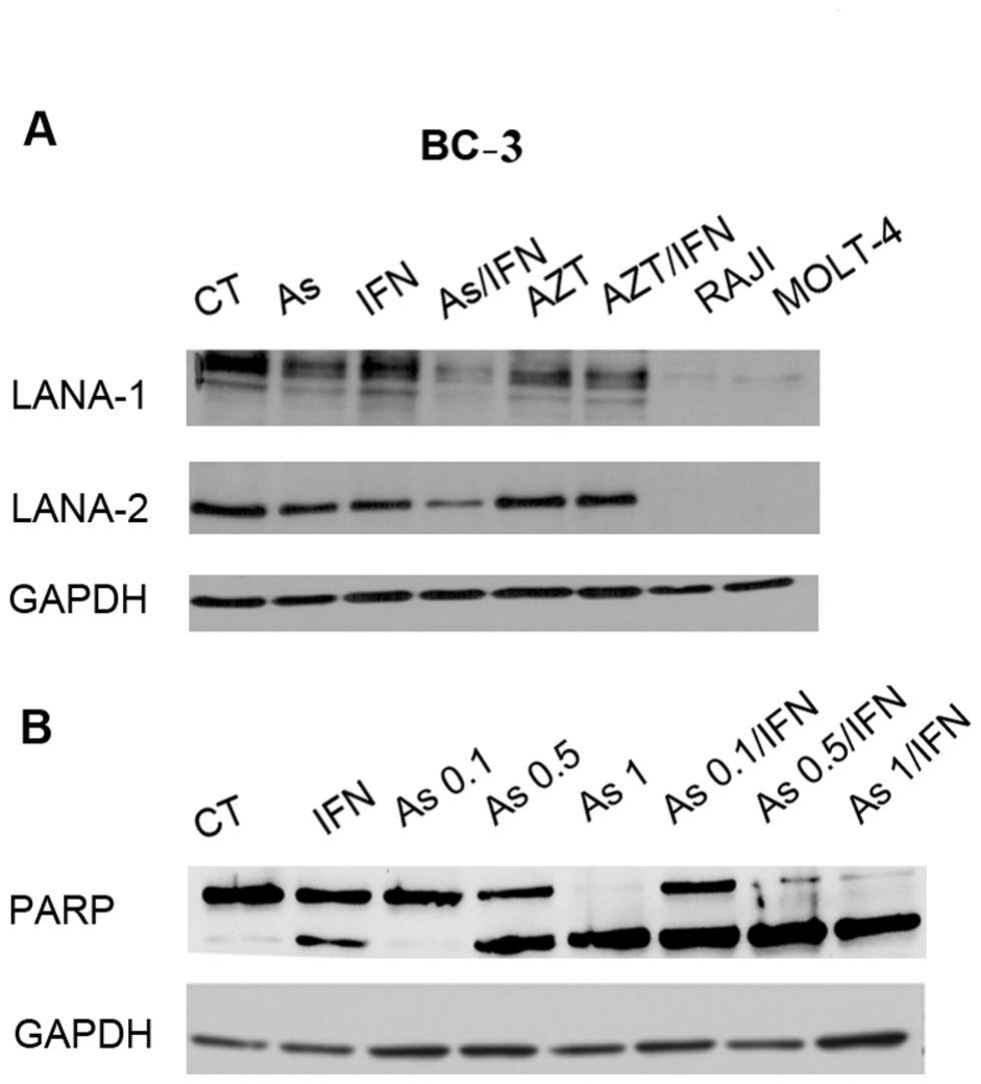
Arsenic/IFN synergistically inhibited expression of latent viral transcripts. Relative expression levels of different samples were calculated by standardization of the amount of target transcript for (A) LANA-1, (B) v-Cyclin or (C) v-FLIP, in a sample to the amount of housekeeping Glucose-6-phosphate dehydrogenase (G6PDH) RNA analyzed in the same sample. In addition, the averages of the normalized control values of Glucose-6-phosphate dehydrogenase (G6PDH) for each sample were used to determine the relative changes in gene expression of the KSHV latency protein LANA-1 by the comparative CT method (2^-ΔΔ^C_T_) [12]. Untreated BC3 and BCBL-1 ascites were used as a calibrator for viral gene expression. The Y-axis represents the relative quantities expressed as percent of the control. The symbol * was used to compare treatment groups to control, while the symbol ‡ was used to compare combination treatments to single treatments. (*, ‡) indicates p< 0.05; (**, ‡‡) indicates p< 0.01; and (***, ‡‡‡) indicates p< 0.001.

In ex-vivo treated malignant ascites-derived BC-3 cells, an important variation in transcript level was noted with single agent treatment with either arsenic or IFN (p<0.001) except for LANA-1 transcript with IFN (Figure 5A). Importantly, arsenic/IFN additively or synergistically decreased the expression of the three viral transcripts to more than 70% of untreated control in malignant ascites-derived BC-3 cells (Figure 5, p<0.001). This decrease in transcript levels of LANA-1 is consistent with its downregulation at the protein level, after single agent treatment with arsenic (Figure 4B). Strikingly, this downregulation was more pronounced with the combination of arsenic and IFN (Figure 4B). Similarly, the viral latent protein LANA-2 was only downregulated with the combination of arsenic/IFN (Figure 4B). This downregulation of latent proteins preceded cell death as evidenced by the persistence of a high percentage of living cells in treated PEL mice (Figure S4F). Conversely, in malignant ascites-derived BCBL-1 cells, arsenic alone or combined with IFN significantly reduced LANA-1 (Figure 5A, p<0.001) and v-cyclin (Figure 5B, p<0.001) expression but had a minimal effect on v-FLIP expression (Figure 5C, p<0.01 and p<0.05). On the other hand, AZT/IFN treatment decreased v-cyclin and v-FLIP expression in BC-3 cells (Figure 5B, p<0.001) and v-cyclin and LANA-1 expression in BCBL-1 cells (Figure 5B, p<0.001). In order to check whether the effect of arsenic and IFN on LANA-1, v-FLIP and v-Cyclin transcript levels was observed at "non-toxic" concentrations, transcripts levels were measured with single concentration of IFN (1000 IU/ml), and varying concentrations of arsenic (0.1, 0.5 and 1 µM) (Figure S5 A-C). The significant decrease in transcripts level was noticed for arsenic concentrations equal to or higher than 0.5 µM. Therefore, in PEL cells, expression of latent KSHV transcripts is inhibited by combination treatments of IFN with arsenic.

Since increases in lytic gene expression are often accompanied by reductions in latent transcripts [68] transcript level of ORF50/RTA lytic gene was assessed (Figure S5D). Both arsenic/IFN and AZT/IFN combinations resulted in a significant increase in ORF50/RTA transcript level. 

## Discussion

In this report, we have demonstrated that the combination of arsenic and IFN delayed ascites development and synergistically prolonged survival of PEL mice. *Ex-vivo*, inhibition of proliferation of PEL cells derived from malignant ascites was associated with induction of apoptosis and significant decrease in the transcript level of KSHV latent proteins. Our results could provide an experimental basis for combined arsenic/IFN treatment of PEL patients. 

The use of IFN alone or in combination with other drugs has held promise for the treatment of several hematological malignancies and solid tumors. Although IFN alone significantly impairs growth of PEL cells *in vitro* [64,69], its use in PEL mice only resulted in minimal increase of survival. Notably, there is at least one report in the literature of a PEL patient who responded to IFN [46,70]. As previously reported, we confirmed that the combination of IFN and AZT induced apoptosis in PEL NOD/SCID mice [44–46]. 

On the other hand, arsenic is known to be a very effective treatment of APL [48,49,51–54–54], and a promising treatment of ATL, particularly when combined with IFN [56–63]. Interestingly, we observed a drastic cooperation of arsenic with IFN in PEL. Indeed, following treatment of PEL mice, ascites formation was impeded and survival was prolonged in a highly synergistic manner between arsenic and IFN. Similarly, in *ex-vivo* treated PEL cells, these two agents synergized to induce apoptosis and inhibit proliferation. This dramatic synergy between arsenic and IFN in KSHV infected PEL cells or PEL mice, is reminiscent of their synergy in HTLV-I infected ATL cells or Tax transgenic ATL mice [57–63]. 

Arsenic alone, or combined to another therapeutic agent against many blood malignancies, is emerging as a potent agent for the eradication of leukemia initiating cells (LIC). For instance, LICs clearance with arsenic alone or combined to ATRA in APL [51], to cytarabine in chronic myeloid leukemia [71], and to IFN in ATL [63] appears to be the main mechanism of leukemia eradication and disease cure in animal models. However, in PEL, our preliminary results indicate that arsenic/IFN treatment did not impair PEL development in serial transplantation assays (data not shown), indicating that the mechanism of action of this combination in PEL is different from other malignancies.

In APL, at the molecular level, arsenic specifically leads to the degradation of the PML/RARα oncoprotein [72], whereas IFN activates the transcription of the PML gene. Although arsenic therapy carries some side effects such as APL blast differentiation, this targeted therapy is significantly less toxic than chemotherapy and combined arsenic/ATRA treatment improved APL survival [73]. In ATL cells, at the molecular level, arsenic/IFN specifically induces proteosomal degradation of the HTLV-1 oncoprotein Tax and reversal of NF-кB activation [58,59,74]. Interestingly, here we show that these two agents synergistically inhibit expression of KSHV latent transcripts. Although v-Cyclin and v-FLIP are transcribed from the same promoter, the v-FLIP coding region is present in a bicistronic messenger, following the v-cyclin coding region. Low et al. have identified an internal ribosome entry site (IRES) preceding the v-FLIP start codon and overlapping the v-cyclin coding region, which allows v-FLIP translation [75]. Yet the dramatic synergy between arsenic and IFN was consistent on all three KSHV latent transcripts (LANA-1, vFLIP and v-Cyc). 

On the other hand, Replication and Transcription Activator (RTA) (also referred to as ORF50), is an immediate-early gene product of KSHV, and plays a critical role in balancing the viral life cycle between latency and lytic replication. RTA has been shown to act as a strong transcription activator for several downstream genes of KSHV. LANA-1 has been shown to block the expression of RTA/ORF50 and to tether the viral episomal DNA to the host chromosomes [76]. Consistent with these studies, our data revealed that the combination of arsenic or AZT with IFN leads to an upregulation of RTA/ORF50 accompanying the downregulation of latent viral transcripts (LANA-1, v-cyclin and v-FLIP) (Figure 5). Finally, induction of RTA/ORF50 and downregulation of latent transcripts may reflect an upstream effect on cohesins and an induction towards the reactivation of KSVH viral replication before triggering cell death and apoptosis as reported [77].

PEL remains of poor prognosis. Novel effective drugs are warranted to improve morbidity and mortality and to reduce the emergence of resistant clones. Our results strongly support the development of a phase II clinical study for the treatment of KSHV associated PEL by the combination of arsenic and IFN.

## Supporting Information

Figure S1 **Histopathology of infiltrated spleen, liver, lung and peritoneum in untreated BC-3 and BCBL-1 PEL-like mice.**
(TIF)

Figure S2 **Arsenic/IFN induces cell growth inhibition and apoptosis in PEL positive but not in KSHV negative cell lines.** (**A**) PEL positive cell lines (BCBL-1, BC-3 and BC-1) and KSHV negative cell lines (RAJI, Jurkat and BL-41) were treated with arsenic 0.1, 0.5, 1μM and IFN (1000), alone, or combinations as indicated. Cell growth (% of control) was assayed in triplicate wells (**B**) Annexin V/PI staining. BC-1 and BL-41 cells were treated for 24h. Histograms represent the percentage of apoptotic cells. Results are plotted as mean ± SD.
(TIF)

Figure S3 **Arsenic/IFN synergistically induced apoptosis of ascites-derived BC3 (left) and BCBL-1 cells (right).** (**A**) Annexin V staining: BC-3 and BCBL-1 ascites were treated for 48h. Representative experiment showing the overlay between the control (blue) and different treatment conditions (green) of FITC-Annexin V flow cytometry charts. (**B**) **TUNEL assay**: BC-3 and BCBL-1 cells derived from PEL ascites were treated for 72h. Representative experiment showing the flow cytometry graphs. Percent of apoptotic cells (TUNEL positive) is indicated on each graph.
(TIF)

Figure S4 **KSHV expression in PEL-derived BC-3 ascites. (A)** Western blot analysis for LANA1 and LANA2 proteins in *ex* *vivo* cultured BC3 ascites for 2 to 11 days as indicated. (**B**) Relative LANA1 transcript expression over actin by RT-PCR in *ex-vivo* cultured BC3 ascites over time. Results are represented as percent of LANA-1 expression for 1 to 9 days. (**C**) Immunocytochemistry (ICC) on BC-3 (Left) and BCBL-1 (right) derived ascites without (upper panel) and with LN-35 rat monoclonal antibody against LANA-1 (lower panel). Viral Protein expression is demonstrated by a finely speckled nuclear pattern with brown staining (Magnification 40X). (D) Immunofluorescence on BC-3 derived ascites without (upper panel) and with LN-35 rat monoclonal antibody against LANA-1 (lower panel). Viral Protein expression is demonstrated by a finely speckled nuclear pattern (Magnification 63X). (**E**) Human CD45 expression in BC3-derived ascites as compared to a negative control (isotype) by flow cytometry. (**F**) Viability of ascites-derived BC-3 cells measured by trypan blue exclusion dye after *in* *vivo* treatment with different dugs. Results are shown as percent of control.
(TIF)

Figure S5 **Arsenic/IFN induced downregulation of latent viral transcripts and upregulation of lytic viral transcript.** Relative expression of LANA-1 (**A**), V-cyclin (**B**), V-FLIP (**C**) after 48h *ex-vivo* treatment with IFN alone, dose dependent concentrations of arsenic alone, or indicated combinations. (**D**) Relative expression of ORF50/RTA after treatment with arsenic, IFN, AZT, arsenic/IFN or AZT/IFN.
(TIF)
