# Supplementary figures and images for: Combination of Arsenic and Interferon-α Inhibits Expression of KSHV Latent Transcripts and Synergistically Improves Survival of Mice with Primary Effusion Lymphomas
Source: PLoS One. 2013 Nov 8;8(11):e79474. doi: 10.1371/journal.pone.0079474 (PMC3826709; doi:10.1371/journal.pone.0079474)

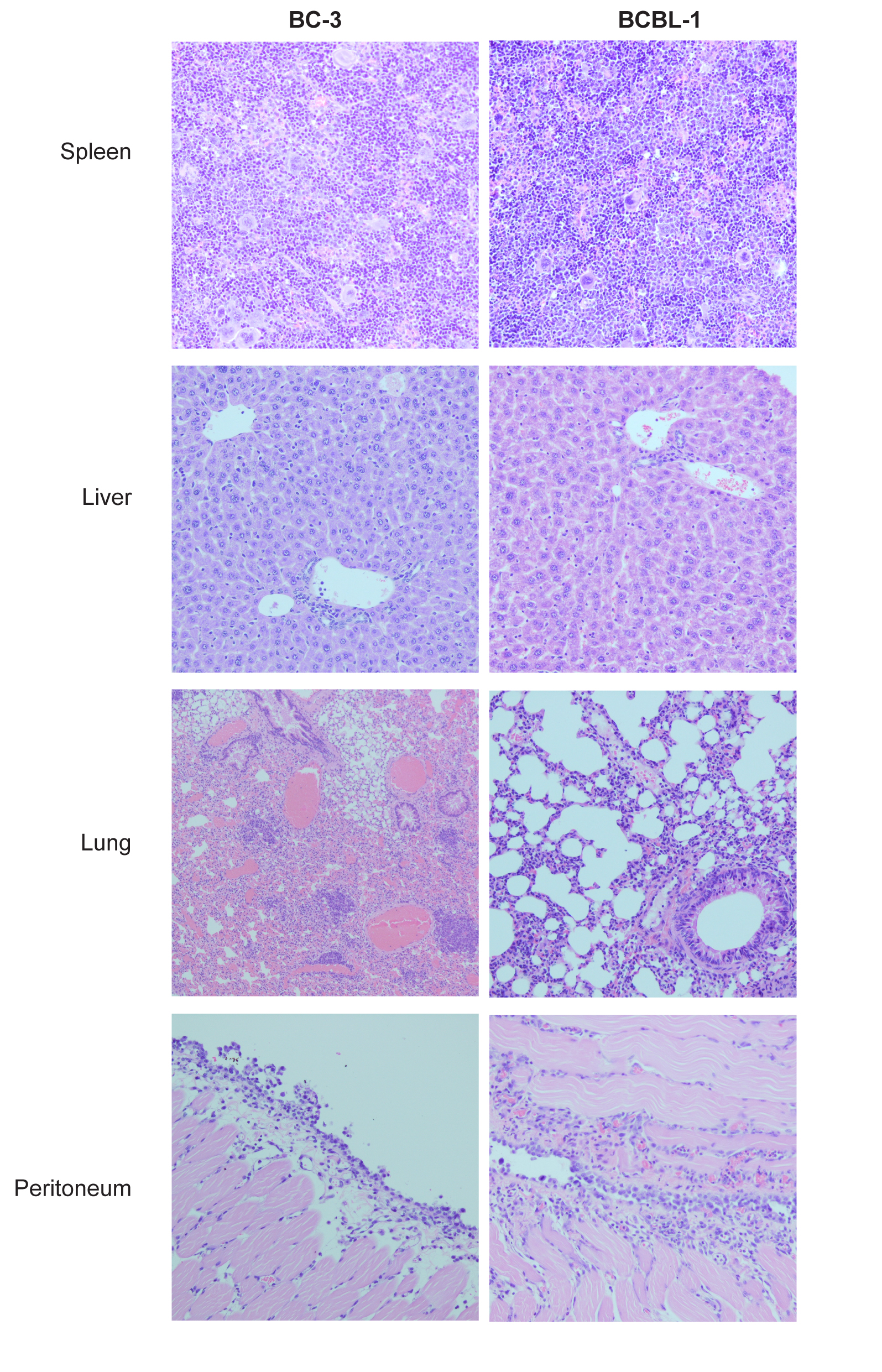

Supplement: Figure S1 — Histopathology of infiltrated spleen, liver, lung and peritoneum in untreated BC-3 and BCBL-1 PEL-like mice. (TIF) [file pone.0079474.s001.tif]

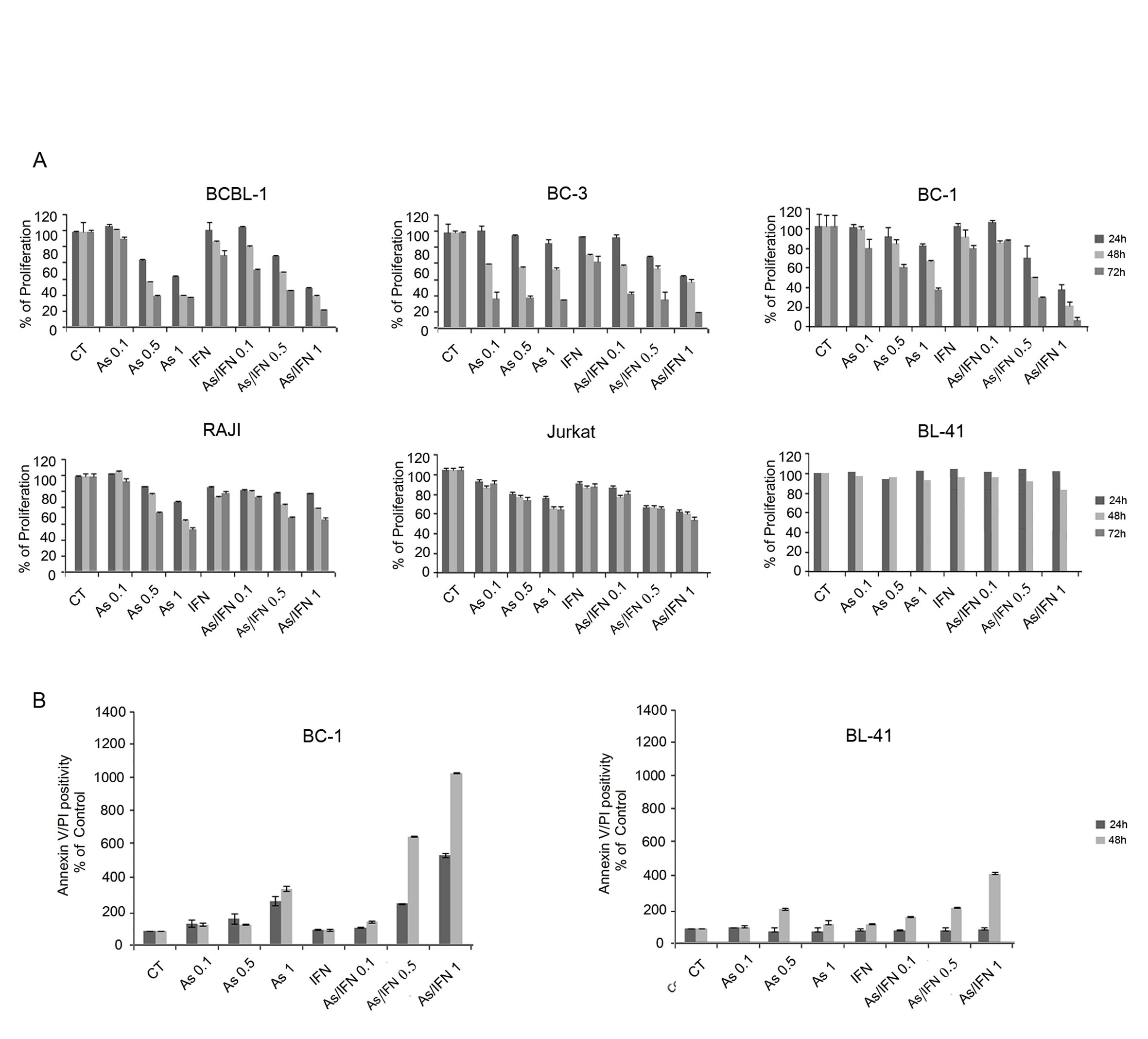

Supplement: Figure S2 — Arsenic/IFN induces cell growth inhibition and apoptosis in PEL positive but not in KSHV negative cell lines. (A) PEL positive cell lines (BCBL-1, BC-3 and BC-1) and KSHV negative cell lines (RAJI, Jurkat and BL-41) were treated with arsenic 0.1, 0.5, 1μM and IFN (1000), alone, or combinations as indicated. Cell growth (% of control) was assayed in triplicate wells (B) Annexin V/PI staining. BC-1 and BL-41 cells were treated for 24h. Histograms represent the percentage of apoptotic cells. Results are plotted as mean ± SD. (TIF) [file pone.0079474.s002.tif]

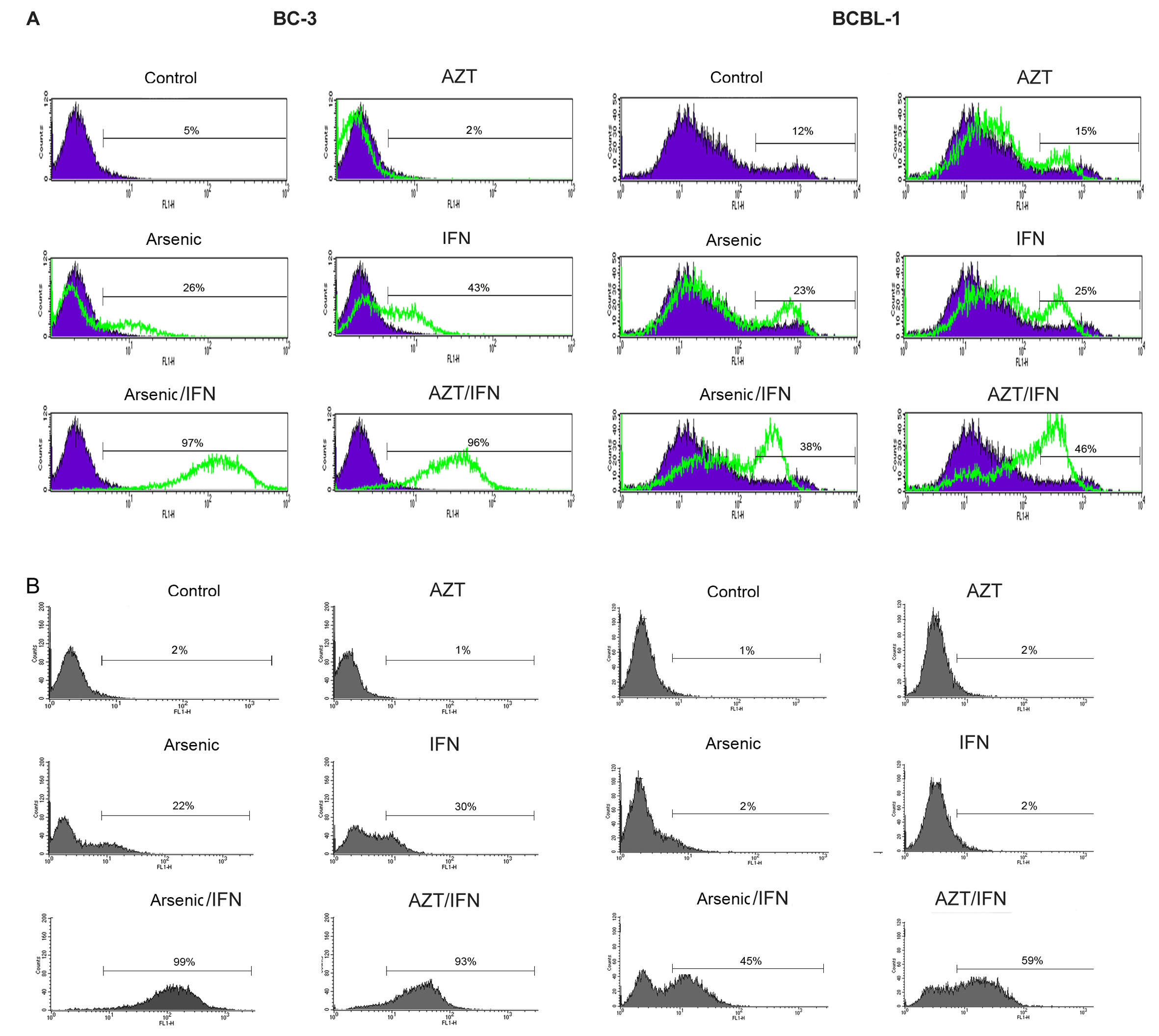

Supplement: Figure S3 — Arsenic/IFN synergistically induced apoptosis of ascites-derived BC3 (left) and BCBL-1 cells (right). (A) Annexin V staining: BC-3 and BCBL-1 ascites were treated for 48h. Representative experiment showing the overlay between the control (blue) and different treatment conditions (green) of FITC-Annexin V flow cytometry charts. (B) TUNEL assay: BC-3 and BCBL-1 cells derived from PEL ascites were treated for 72h. Representative experiment showing the flow cytometry graphs. Percent of apoptotic cells (TUNEL positive) is indicated on each graph. (TIF) [file pone.0079474.s003.tif]

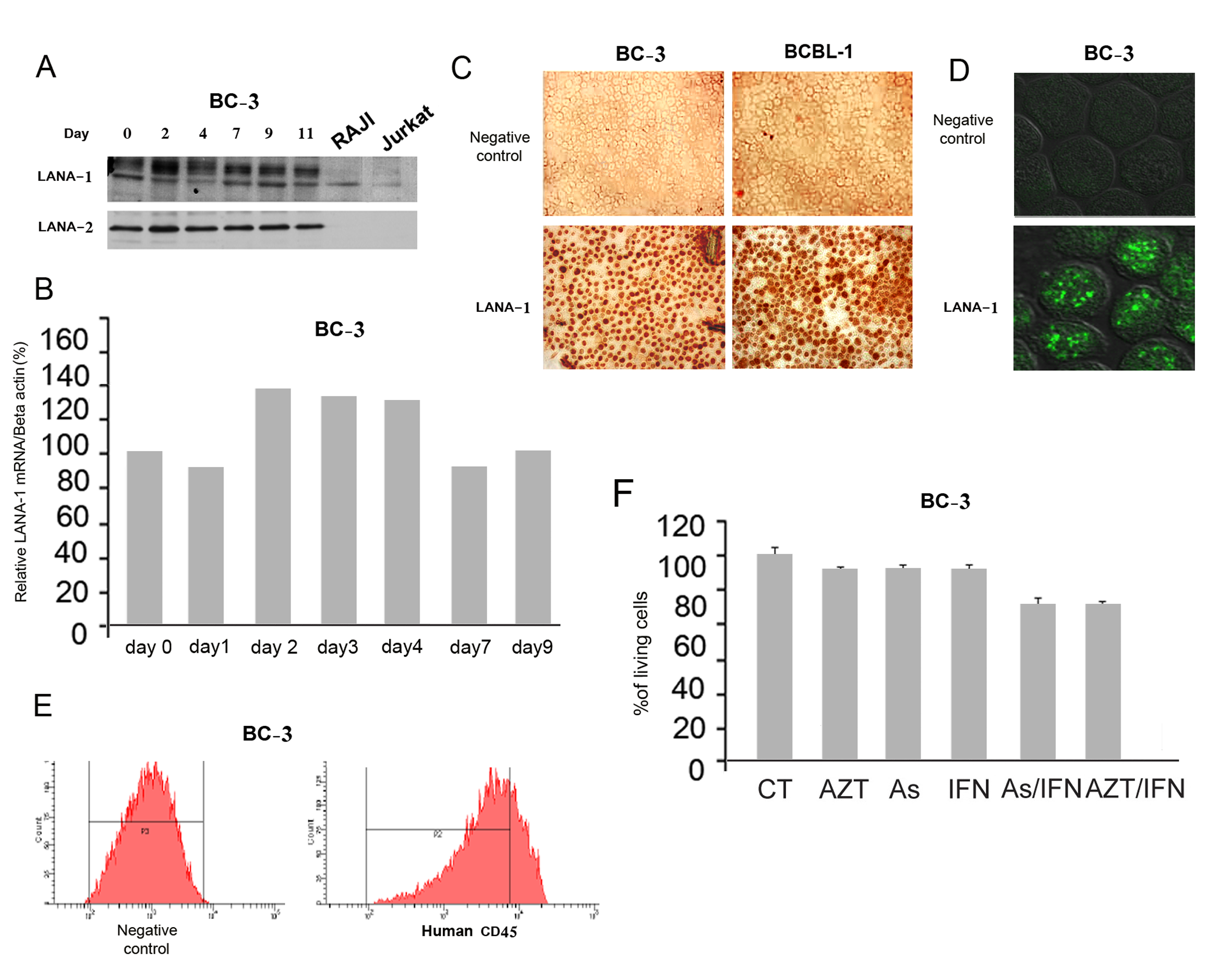

Supplement: Figure S4 — KSHV expression in PEL-derived BC-3 ascites. (A) Western blot analysis for LANA1 and LANA2 proteins in ex vivo cultured BC3 ascites for 2 to 11 days as indicated. (B) Relative LANA1 transcript expression over actin by RT-PCR in ex-vivo cultured BC3 ascites over time. Results are represented as percent of LANA-1 expression for 1 to 9 days. (C) Immunocytochemistry (ICC) on BC-3 (Left) and BCBL-1 (right) derived ascites without (upper panel) and with LN-35 rat monoclonal antibody against LANA-1 (lower panel). Viral Protein expression is demonstrated by a finely speckled nuclear pattern with brown staining (Magnification 40X). (D) Immunofluorescence on BC-3 derived ascites without (upper panel) and with LN-35 rat monoclonal antibody against LANA-1 (lower panel). Viral Protein expression is demonstrated by a finely speckled nuclear pattern (Magnification 63X). (E) Human CD45 expression in BC3-derived ascites as compared to a negative control (isotype) by flow cytometry. (F) Viability of ascites-derived BC-3 cells measured by trypan blue exclusion dye after in vivo treatment with different dugs. Results are shown as percent of control. (TIF) [file pone.0079474.s004.tif]

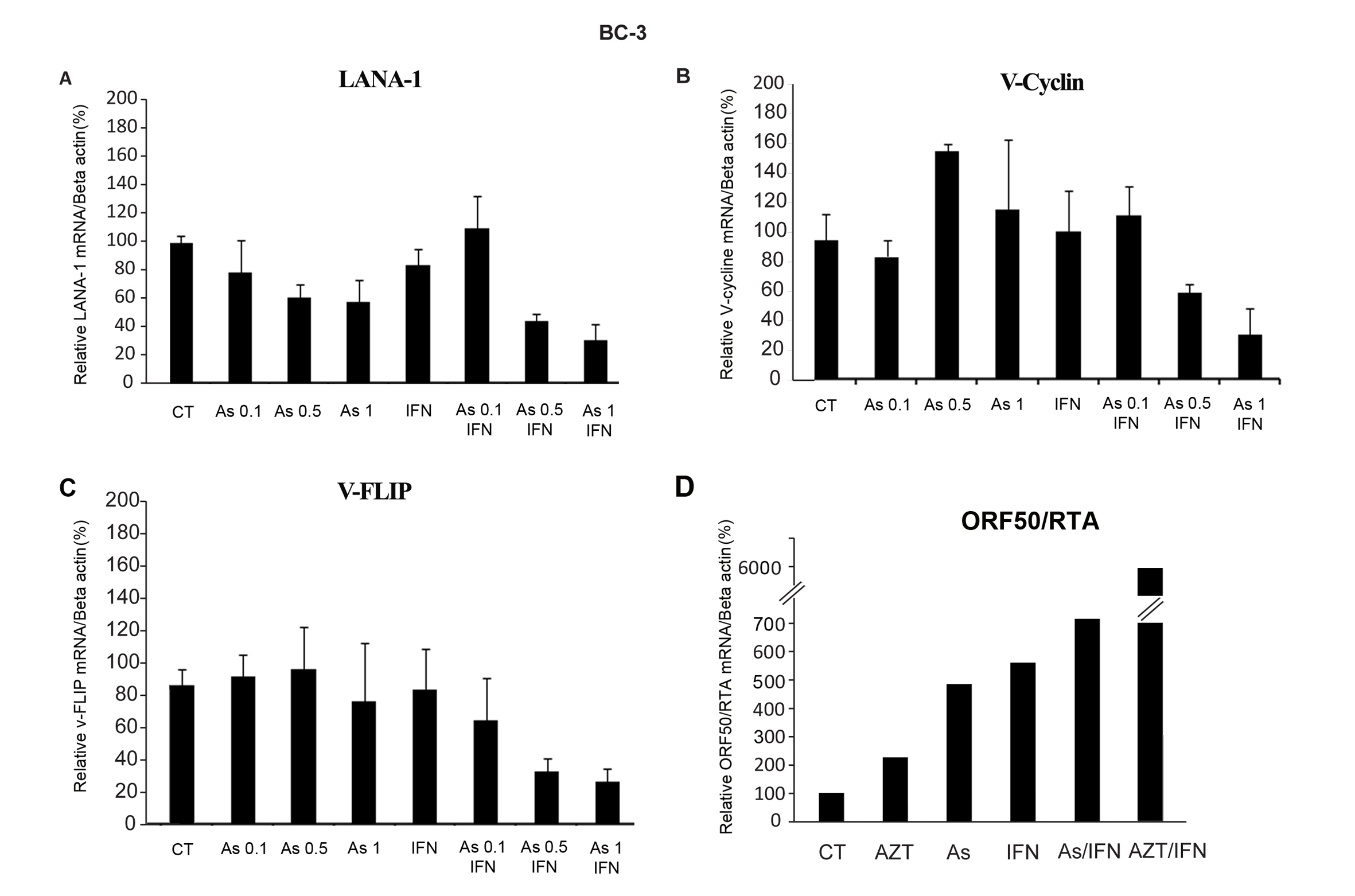

Supplement: Figure S5 — Arsenic/IFN induced downregulation of latent viral transcripts and upregulation of lytic viral transcript. Relative expression of LANA-1 (A), V-cyclin (B), V-FLIP (C) after 48h ex-vivo treatment with IFN alone, dose dependent concentrations of arsenic alone, or indicated combinations. (D) Relative expression of ORF50/RTA after treatment with arsenic, IFN, AZT, arsenic/IFN or AZT/IFN. (TIF) [file pone.0079474.s005.tif]
